# Targeting the MAPK7/MMP9 axis for metastasis in primary bone cancer

**DOI:** 10.1038/s41388-020-1379-0

**Published:** 2020-07-13

**Authors:** Darrell Green, Heather Eyre, Archana Singh, Jessica T. Taylor, Jason Chu, Lee Jeys, Vaiyapuri Sumathi, Aman Coonar, Doris Rassl, Muhammad Babur, Duncan Forster, Saba Alzabin, Frida Ponthan, Adam McMahon, Brian Bigger, Tristan Reekie, Michael Kassiou, Kaye Williams, Tamas Dalmay, William D. Fraser, Katherine G. Finegan

**Affiliations:** 1 Norwich Medical School, University of East Anglia, Norwich, UK; 2 Faculty of Biology Medicine and Health, University of Manchester, Manchester, UK; 3 Digital Biology, Earlham Institute, Norwich, UK; 4 Orthopaedic Oncology, The Royal Orthopaedic Hospital, Birmingham, UK; 5 Musculoskeletal Pathology, The Royal Orthopaedic Hospital, Birmingham, UK; 6 Thoracic Surgery, The Royal Papworth Hospital, Cambridge, UK; 7 Pathology, The Royal Papworth Hospital, Cambridge, UK; 8 Wolfson Molecular Imaging Centre, University of Manchester, Manchester, UK; 9 Epistem Limited, Manchester, UK; 10 School of Chemistry, University of Sydney, Sydney, Australia; 11 School of Biological Sciences, University of East Anglia, Norwich, UK; 12 Clinical Biochemistry, Norfolk and Norwich University Hospital, Norwich, UK; 13 Diabetes and Endocrinology, Norfolk and Norwich University Hospital, Norwich, UK

**Keywords:** Bone cancer, Mechanisms of disease, Transcriptomics

## Abstract

Metastasis is the leading cause of cancer-related death. This multistage process involves contribution from both tumour cells and the tumour stroma to release metastatic cells into the circulation. Circulating tumour cells (CTCs) survive circulatory cytotoxicity, extravasate and colonise secondary sites effecting metastatic outcome. Reprogramming the transcriptomic landscape is a metastatic hallmark, but detecting underlying master regulators that drive pathological gene expression is a key challenge, especially in childhood cancer. Here we used whole tumour plus single-cell RNA-sequencing in primary bone cancer and CTCs to perform weighted gene co-expression network analysis to systematically detect coordinated changes in metastatic transcript expression. This approach with comparisons applied to data collected from cell line models, clinical samples and xenograft mouse models revealed mitogen-activated protein kinase 7/matrix metallopeptidase 9 (MAPK7/MMP9) signalling as a driver for primary bone cancer metastasis. RNA interference knockdown of *MAPK7* reduces proliferation, colony formation, migration, tumour growth, macrophage residency/polarisation and lung metastasis. Parallel to these observations were reduction of activated interleukins *IL1B*, *IL6*, *IL8* plus mesenchymal markers *VIM* and *VEGF* in response to *MAPK7* loss. Our results implicate a newly discovered, multidimensional MAPK7/MMP9 signalling hub in primary bone cancer metastasis that is clinically actionable.

## Introduction

Primary bone cancer (PBC) is the third most common solid childhood cancer with 52,000 new cases per year worldwide [1]. PBC arises at the ends of long bones, usually on either side of the knee/pelvis. PBC includes several molecular subtypes, of which osteosarcoma is the most common. Osteosarcomas can occur in adults, but these are usually secondary to radiation exposure or Paget’s disease of bone [2, 3]. Major driver mutations for osteosarcoma include tumour protein p53 (*TP53*) and RB transcriptional corepressor 1 (*RB1*) structural variants that trigger chromothripsis [4–7]. Around 25% of patients present with detectable metastasis (85% with lung metastases, 15% with skeletal metastases). Five-year survival with metastatic/relapsed osteosarcoma is 15% [1, 8]. Survival rates have not changed for more than four decades. A better understanding of the molecular and cellular mechanisms that underpin spread is urgent.

Metastasis is the leading cause of cancer-related death. This multistage and complex process requires metastatic cells to shed into the local vasculature, survive circulation, extravasate at distant sites and proliferate. Metastasis involves contribution from both tumour cells and tumour stroma. The early stages of metastasis are relatively efficient. Post-extravasation stages, that is, colonisation, are critical in determining metastatic outcome [9]. It is largely accepted that cancer arises from linear Darwinian evolution involving competing subclones within a single tumour that eventually culminates in lethal clones with metastatic capability. Evidence suggests that metastatic dissemination may occur early where cells from incipient, low-density lesions display more stemness and metastatic tendency than cells from proliferative, high-density tumours [5, 9]. Analysis of secondary lesions to elucidate molecular properties of spread is hampered by the extreme difficulty in obtaining samples of metastatic disease because of a lack of surgical intervention at that clinical stage. Circulating tumour cells (CTCs) provide an alternative less invasive approach where samples may be accessed throughout the disease course plus reveal mechanisms of spread with the potential to identify novel therapeutic strategies. CTC-based studies in breast, prostate, and lung cancer show evidence of high *WNT* signalling plus high haemoglobin subunit beta (*HBB*) to support circulatory survival [10]. CTC clustering causes demethylation of POU class 5 homeobox 1 (*POU5F1*), SRY box 2 (*SOX2*), nanog homeobox (*NANOG*) and SIN3 transcription regulator family member A (*SIN3A*), all genes paralleling stemness [11]. It still remains unclear how CTCs are released from tumours. Studies suggest that interaction between tumour cells and immune cells in the tumour microenvironment influences metastatic progression [12, 13]. Immunotherapies that target tumour stroma interactions instead of tumours directly have shown efficacy in several cancers shedding light on the possible treatment of PBC [14].

Reprogramming the transcriptomic landscape in tumour cells and in the tumour stroma is a metastatic hallmark, but detecting underlying master regulators that drive pathological gene expression is a key challenge, especially in childhood cancer. Here we used an integrated analytical approach that combines whole tumour plus single CTC RNA-sequencing of patient samples (Supplementary File 1) to search for PBC metastasis master regulators. A co-expression network was built on all genes using a cut-off mean (transcripts per millions (TPM) >5). We searched for gene modules that were enriched for differentially expressed (DE) genes. Modules enriched for DE genes were used to reveal metastasis-associated genes. The functions of metastasis-associated genes were enriched to determine the importance of these genes in PBC spread. Using these clinical datasets as a guide, we generated a xenograft mouse model to mechanistically reveal a novel tumour cell–immune cell interaction that drives PBC metastasis to the lungs.

## Results

### Recurrent *HH*, *FGFR* and *IGF* in whole tumours

**Fig. 1:**
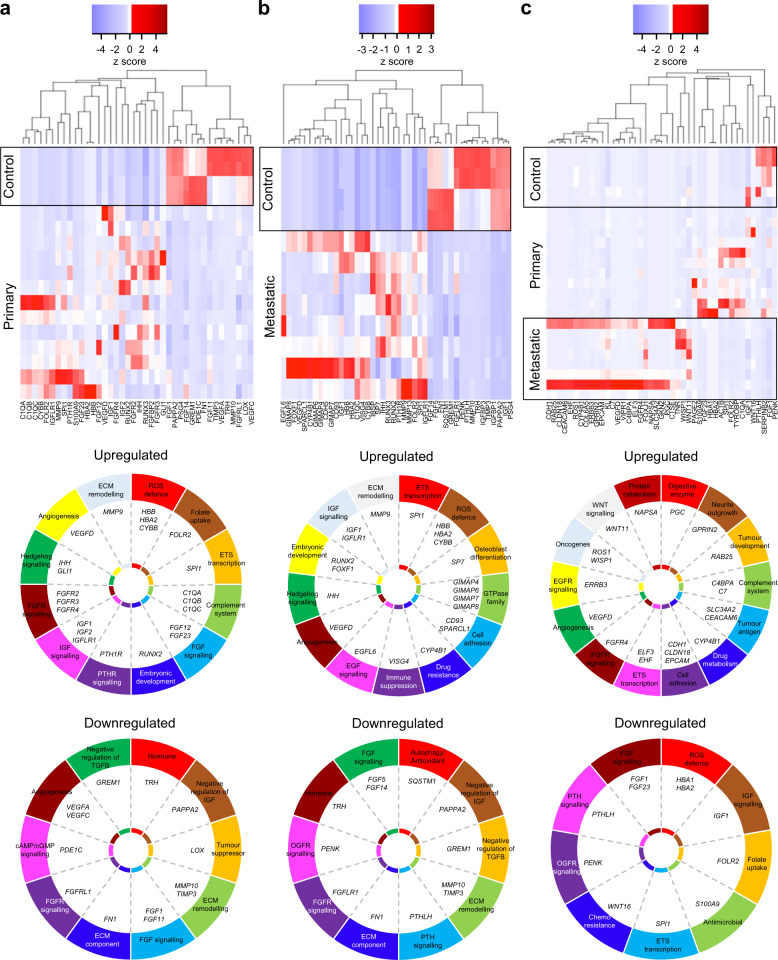
Heat map-based hierarchical cluster analysis of DE genes (*x*-axis) across tissue type (*y*-axis). *Z*-score refers to high (red) and low (blue) gene expression using normalised values when compared to the mean of total sequencing reads. Pie charts below each heat map visually represent altered genes/pathways. **a** Control bone versus primary tumour. **b** Control bone versus metastatic lesion. **c** Primary tumour versus metastatic lesion. There were few differences in gene expression between MAP-treated and non-MAP-treated patients. Patients are presented as one cohort, which will also include endogenous genetic heterogeneity. Each transcript presented has passed log_2_ fold change ≥2, *p* ≤0.05 and FDR ≤ 5% parameters.

An observation of immediate therapeutic significance was the increased expression of hedgehog (*HH*), RUNX family transcription factor 2 (*RUNX2*), fibroblast growth factor receptor (*FGFR*) and insulin-like growth factor (*IGF*) in whole tumours when compared to controls (Fig. 1a, b). These data are consistent with our own and others’ observations, including the recent report of *IGF1* amplification in 14% of osteosarcomas [1, 15, 16], although *IGF1* plays less of a role for driving primary tumour to metastatic tumour gene expression (Fig. 1c). Given the poor osteosarcoma prognosis and lack of treatment progress, our findings provide a reason for exploring the efficacy of targeting these pathways as first-line treatment. Sonidegib to target *HH*, Lenvatinib to target *FGFR* and Cixutumumab, Dalotuzumab and Robatumumab to target *IGF* have shown promising antineoplastic activity in other cancers [17–19].

### Induction therapy activates folate receptor beta

Induction therapy for osteosarcoma in the United Kingdom comprises high-dose methotrexate, doxorubicin and cisplatin (MAP). We showed that the transcript for the cellular receptor for folic acid uptake, folate receptor beta (*FOLR2*), is upregulated in osteosarcoma exposed to MAP (Fig. 1a, b). These data infer a biological mechanism for chemoresistance as methotrexate will be less obstructive to neoplastic folic acid metabolism.

### Alternative splicing in several transcripts

Alternative splicing events are categorised as skipped exon, retained intron, alternative 5′ splice site, alternative 3′ splice site and mutually exclusive exon. We report events in several transcripts not previously implicated in osteosarcoma (Supplementary Fig. 1). These transcripts include 2-oxoglutarate and iron-dependent oxygenase domain containing 2 (*OGFOD2*), autophagy-related 4D cysteine peptidase (*ATG4D*), tropomyosin 1 (*TPM1*), transmembrane protein 218 (*TMEM218*), copine 1 (*CPNE1*) and WW domain binding protein 1 (*WBP1*) (Supplementary Fig. 1). These DE transcripts harbour four of five alternative splicing events.

### Single osteosarcoma CTCs

**Fig. 2:**
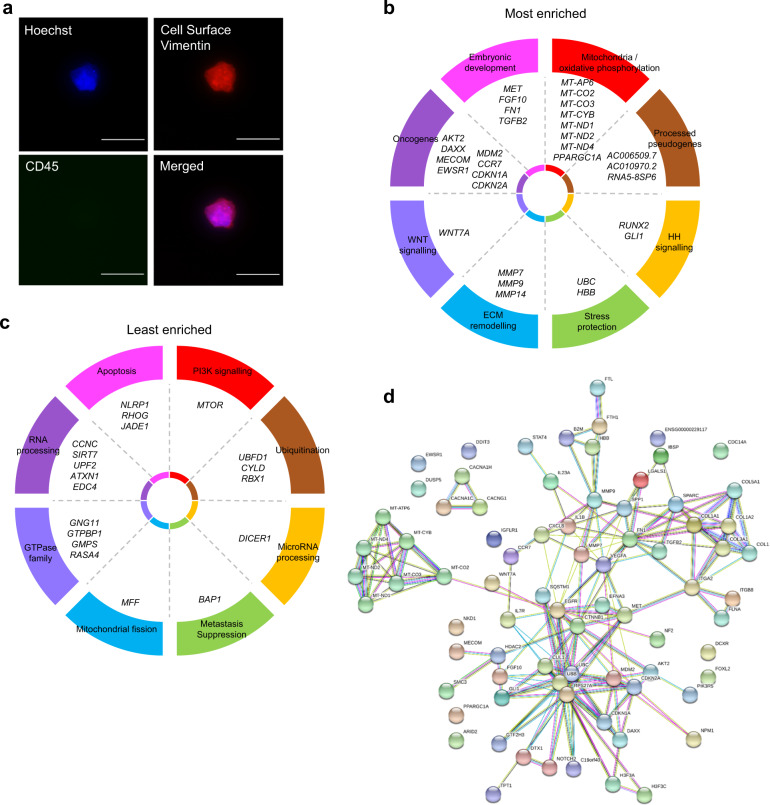
Single cell RNA sequencing of circulating tumour cells. **a** CTCs are positive for cell surface vimentin and negative for CD45. Scale bar is 50 μM. **b** Pie chart visually represents the most enriched transcripts. **c** Pie chart visually represents the least enriched transcripts. **d** Gene–gene connections at high confidence (scores between 0.7 and 0.9). Line colour connecting genes indicate the known and predicted interactions. Blue lines represent data from curated databases. Pink lines represent data from experiments. Green lines represent gene neighbourhoods. Black lines represent co-expressed genes.

We achieved <500,000 mapped reads in single CTCs (Fig. 2a) and >30 million mapped reads in whole tumours, so it was inappropriate to directly compare DE genes. We performed numerical expression plus enrichment analysis to intersect the dataset between primary and secondary tumours (Supplementary File 2). There was abundance of mitochondrial gene expression, including mitochondrially encoded cytochrome c oxidase I, II and III (*MT-CO1*, *2*, *3*), mitochondrially encoded NADH:ubiquinone oxidoreductase core subunits 1–4 (*MT-ND1*, *2*, *3*, *4*) and mitochondrially encoded cytochrome b (*MT-CYB*) (Fig. 2b). These transcripts are central to oxidative phosphorylation. Consistent with other cancer types, there was abundance of stress tolerance with expression of *HBB* and ubiquitin C (*UBC*) (Fig. 2b). There were markers of stemness and embryonic activation with expression of MET proto-oncogene, receptor tyrosine kinase (*MET*), fibroblast growth factor 10 (*FGF10*), fibronectin 1 (*FN1*), transforming growth factor beta 2 (*TGFB2*) and *RUNX2* (Fig. 2b). There was also an abundance of collagen-associated transcripts (Fig. 2b). There was a low expression of mitochondrial fission factor (*MFF*), transcripts for RNA processing, including cyclin C (*CCNC*), sirtuin 7 (*SIRT7*), enhancer of mRNA decapping 4 (*EDC4*) and dicer 1, ribonuclease III (*DICER1*) (Fig. 2c). There was a low transcript number for BRCA1-associated protein 1 (*BAP1*), which when highly expressed suppresses metastasis (Fig. 2c) [20]. STRING analysis showed a functional interaction between all transcripts (Fig. 2d).

### *CYP4B1*, *FGFR4* and *ETS* transcription factors in secondary tumours

**Fig. 3:**
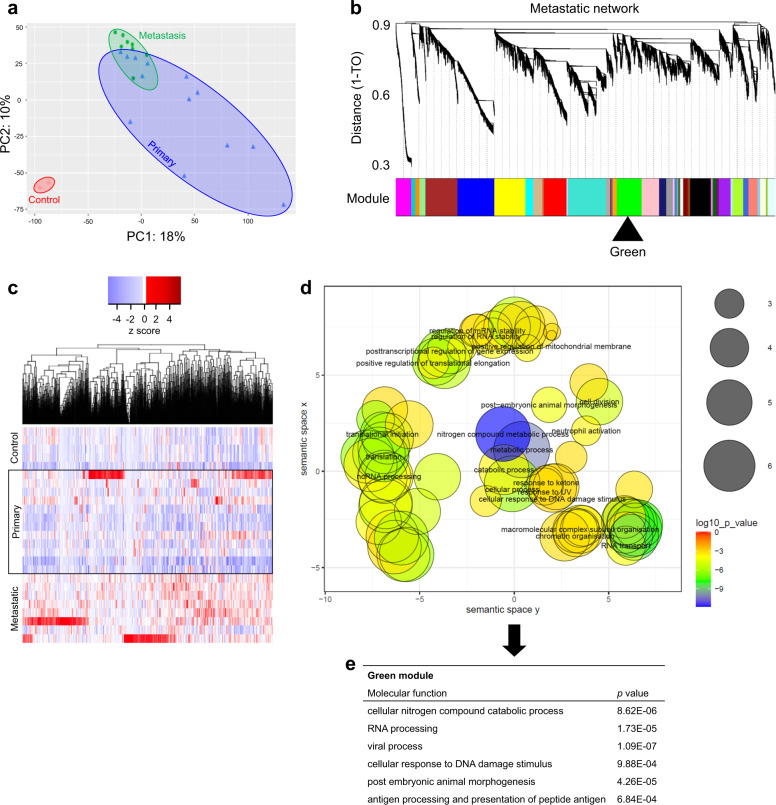
Weighted gene co-expression network analysis determines metastatic drivers. **a** Biplot principal component analysis (PCA) shows groups along the PC1 axis that correspond to primary (blue triangles) and metastatic (green circles) PBC plus controls (red crosses). **b** WGCNA cluster dendrogram on all samples groups genes into distinct driver modules. Co-expression distance (TO, topology overlap) between genes (*y*-axis) and to genes (*x*-axis). Gene modules are colour coded. We selected the Green module where *E2F1* is a hub gene for further analysis because of its relationship to *TP53* and that *MMP9* was a component of the module. **c** Heat map-based hierarchical cluster analysis of the Green module show clear and distinct expression patterns between tissue types. *Z*-score refers to high (red) and low (blue) gene expression using normalised values when compared to the mean of total sequencing reads. **d** GO analysis using REVIGO [52] scatterplot visualisation shows the cluster representatives in a two-dimensional space derived by applying multidimensional scaling to a matrix of the GO terms’ semantic similarities. Bubble colour indicates *p* value. Bubble size indicates the frequency of the GO term in the underlying gene ontology annotation GO term database. **e** Molecular functions significantly affected.

Principal component analysis (PCA) showed grouping between controls, primary tumours and metastases (Fig. 3a). PCA demonstrates the transcriptional trajectory of metastatic progression (Fig. 3a). Gene expression differences included upregulated drug metabolism via cytochrome P450 family 4 subfamily B member 1 (*CYP4B1*) (Fig. 1c). Metastases showed cell adhesive properties via cadherin 1 (*CDH1*), claudin 18 (*CLDN18*) and epithelial cell adhesion molecule (*EPCAM*) (Fig. 1c). There was abundance of fibroblast growth factor receptor 4 (*FGFR4*), Erb-b2 receptor tyrosine kinase 3 (*ERBB3*) and E74-like ETS transcription factor 3 (*ELF3*) expression (Fig. 1c).

### WGCNA discriminates metastasis

Patterns of genes in tissue types can be identified by weighted gene co-expression network analysis (WGCNA). WGCNA is an unsupervised and unbiased analysis that identifies genes with similar expression patterns across samples and assigns correlated genes to distinct co-expression modules [21]. In contrast to standard analysis for network analysis such as cytoscape based approaches, WGCNA seeks to identify higher-order relationships among genes by transforming gene expression profiles into functional co-expressed gene modules. Within groups of highly co-expressed genes or ‘modules’ that comprise core functional units of transcriptional networks, WGCNA identifies central genes connecting the modules termed ‘hubs’. This analysis alleviates several testing problems that are inevitable in standard gene centric methods, making WGCNA a powerful tool in cancer studies [22]. Based on a mean gene expression value of TPM > 5 across patient samples, 19,913 genes were selected for WGCNA. These genes produced 41 co-expression modules comprising 16,369 genes (3544 genes were filtered because they do not cluster to any module) (Fig. 3b and Supplementary File 3). For each of the 41 modules, we identified a hub gene (Supplementary File 3). We examined hubs likely to be involved in metastasis by searching for modules that were enriched for DE genes (control vs. primary tumour, control vs. metastasis, primary tumour vs. metastasis). Twenty-six modules were enriched for DE genes (Fisher’s exact test *p* ≤ 0.05). Heat maps based on normalised TPM values of these 26 modules showed different expression patterns in PBC metastasis to the lung. We selected the Green module (Fig. 3c and Supplementary File 3) where E2F transcription factor 1 (*E2F1*) was the hub gene because *E2F1* mediates *TP53*-dependent apoptosis. This pathway is critical for the current study because of the *TP53*^*−/−*^ driver mutation described earlier [23].

All 1045 genes in the Green module were subject to gene ontology (GO) analysis (Fig. 3d) to show spatial representation of enriched GO terms plusmolecular functions significantly affected (Fig. 3e). Within these analyses, we observed matrix metallopeptidase 9 (*MMP9*) as a candidate pro-metastatic gene. We had also noted *MMP9* as a highly expressed gene in our previous analyses (Figs. 1, 2 and normalised data on GEO), so we selected *MMP9* for further investigation. The other 25 modules were not explored further here, but are freely available in Supplementary File 3.

### MAPK7 is an *MMP9* master regulator and drives lung metastasis in vivo

**Fig. 4:**
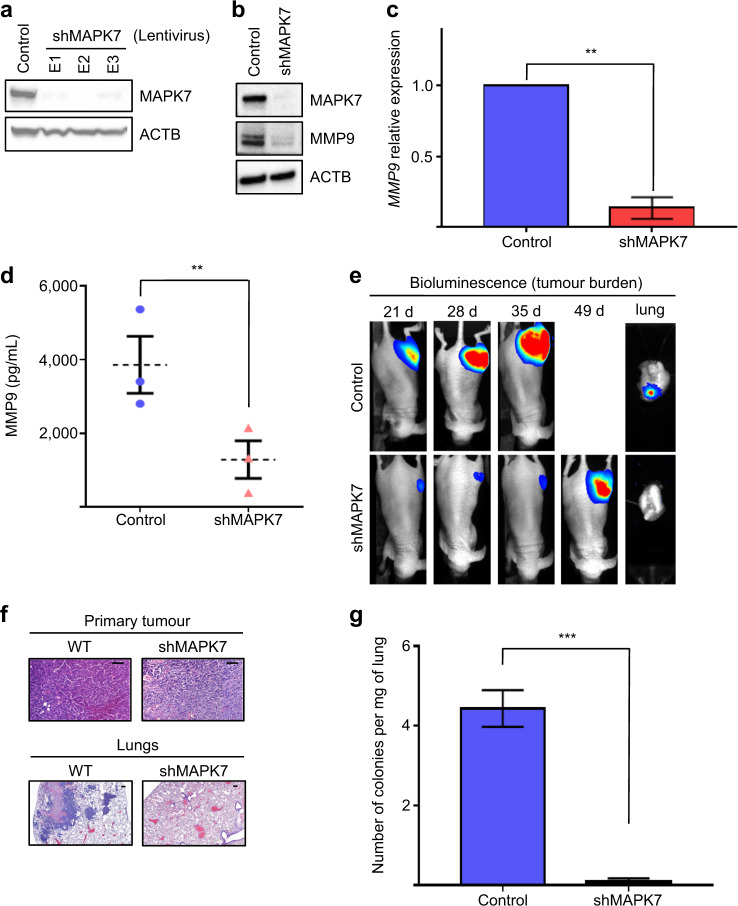
MAPK7 is an MMP9 master regulator and drives lung metastasis in vivo. **a** Immunoblot analysis of MAPK7 expression in 143B cells demonstrating knockdown efficiency of shMAPK7 lentiviral preparations E1, E2 and E3. E2 induced the greatest decrease in MAPK7. E2-mediated MAPK7 cells were used for the rest of the study and are referred to as shMAPK7 cells hereafter. **b** Immunoblot analysis of MMP9 demonstrating loss of MMP9 expression following MAPK7 knockdown in 143B cells. **c** qPCR analysis showing MAPK7 knockdown induces a significant decrease in *MMP9* mRNA. *MMP9* mRNA levels were normalised to *PGK1* mRNA. **d** ELISA analysis of culture media demonstrates that loss of MAPK7 significantly reduces MMP9 secretion by 143B cells. **e** Bioluminescence imaging (BLI) to measure tumour burden in mice implanted intrafemorally with control and shMAPK7 143B cells. Tumours derived from shMAPK7 cells have delayed growth compared to control and display no detectable metastatic spread to the lung (absence of BLI signal in lungs of animals harbouring shMAPK7 143B tumours). **f** Tumour H&E stain from control and shMAPK7 tumours and lungs. **g** Lung clonogenic assay to detect micro metastatic spread to the lung. Lungs from mice harbouring shMAPK7 tumours had virtually no lung clonogenicity (*p* ≤ 0.001). Representative images are used to describe data collected from 12 mice per group. Data are mean ± SD of three biological replicates. **p ≤ 0.05, ***p ≤ 0.001.

MMP9 is a prognostic marker for several cancers, with several studies showing its role in angiogenesis, extracellular matrix and surface receptor cleavage [24–26]. MMP9 inhibitor drugs have had limited success in patient trials [27]. One explanation for MMP9 drug failure is that targeting the catalytic component of MMP9 is insufficient for effect. We asked whether targeting the *MMP9* transcript preventing protein translation may show improved outcomes, so we opted to test the *MMP9* upstream regulator mitogen-activated protein kinase 7 (MAPK7), that is, our goal was to target a master regulator to ‘action’ the ‘unactionable’ MMP9. Supporting this experimental strategy was that MAPK7 also has roles in metastatic cancer [28–33], so we would likely ‘hit’ several other genes/pathways as well as *MMP9*. We cloned highly metastatic human 143B cells with stably expressed short hairpin RNA (shRNA) to suppress *MAPK7* (shMAPK7) (Fig. 4a, b), which had no impact on proliferation in vitro (Supplementary Fig. 2a). To monitor growth of the primary tumour plus tumour dissemination to the lungs, we used luciferase-tagged cells, which also had no impact on proliferation in vitro (Supplementary Fig. 2b). Control and shMAPK7 luciferase-tagged cells displayed comparable, constitutive luciferase activity and bioluminescence signal directly correlated to tumour size in vivo (Supplementary Fig. 2c). We engrafted transfected cells into the femur of immunocompromised mice and tracked metastatic colonisation in the lungs [34]. *MAPK7*-deficient tumours were grown to the same size as control tumours before being tested for metastatic potential and lung clonogenicity, ensuring we compared ‘like for like’. Metastatic cells harbouring shMAPK7 showed significantly reduced *MMP9* transcript and MMP9 protein expression (Fig. 4c, d). shMAPK7 tumour growth was markedly slower plus cells showed significantly reduced ability to colonise the lungs (Fig. 4e). Lung metastases were undetectable by haematoxylin and eosin (H&E) staining in mice harbouring shMAPK7 tumours (Fig. 4f). Lung clonogenicity, which can be used to detect micrometastases undetectable by H&E staining, showed practically no spread to the lung from shMAPK7 tumours (shMAPK7 = 0.092 colonies per mg, controls = 4.43 colonies per mg of lung, *p* ≤ 0.001) (Fig. 4g) or any other organ (data not shown). These data show that MAPK7 is a master regulator of *MMP9* expression, and reduction of this signalling axis inhibits spread to the lungs.

### MAPK7/MMP9 signalling localises to the invasive margin

**Fig. 5:**
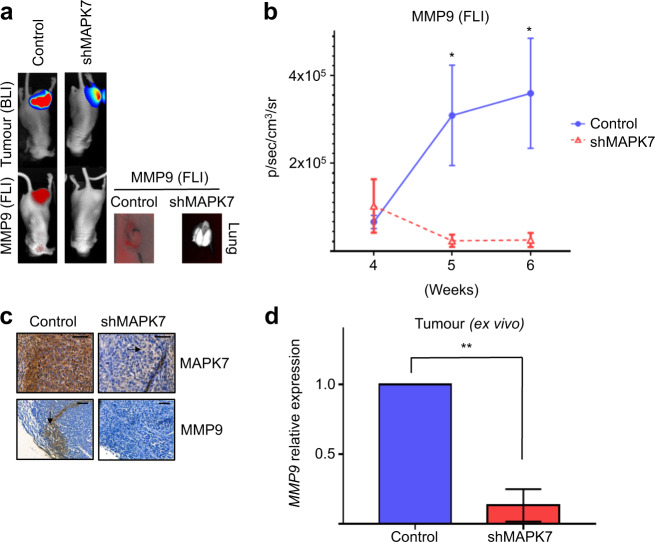
MAPK7/MMP9 signalling localises to the invasive margin. **a** Fluorescence imaging to detect active MMP9 in tumours in vivo. Tumours lacking MAPK7 had no detectable MMP9 activity. Images are from size-matched control and shMAPK7 tumours. **b** Quantified FLI signal in tumours over time. FLI signal indicative of MMP9 activity increases over time in control, but not shMAPK7 tumours. **c** IHC analysis of tumour biopsies. shMAPK7 tumours display marked reduction in MAPK7 expression, but still display MAPK7-positive cells in the stroma (arrow). MMP9 expression was observed at the leading edge of control tumours (arrow), but was undetectable in shMAPK7 tumour biopsies. Scale bar is 100 μM. **d** qPCR analysis of ex vivo tumour lysates. shMAPK7 tumours have significantly less *MMP9* mRNA expression. *MMP9* mRNA levels were normalised to *PGK1* (*p* ≤ 0.001). Representative images are used to describe data collected from 12 mice per group. Data are mean ± SD of three biological replicates. *p ≤ 0.05, **p ≤ 0.001.

Metastasis is independent of tumourigenesis, which is mostly driven by tumour growth. Metastasis can be defined by other features, including invasiveness and colonisation, so we next addressed the MAPK7/MMP9 signalling origin. We used in vivo fluorescence imaging using an MMP9 substrate that fluoresces upon proteolytic cleavage [35]. Fluorescence signal indicative of active MMP9 laterally increased with tumour growth in controls (Fig. 5a, b). Tumour cells harbouring shMAPK7 showed significantly reduced fluorescence signal in both primary tumour and metastases (Fig. 5a, b). We verified fluorescence imaging by analysing MMP9 expression in tumour lysates plus gross histology. MMP9 signal was mostly localised to the tumour edge, that is, the invasive margin (Fig. 5c, arrow lower panel). shMAPK7 tumours displayed MAPK7 in stromal regions only (Fig. 5c, arrow upper panel). Tumours lacking functional *MAPK7* showed MMP9 protein loss (Fig. 5c, d). These data show that MAPK7/MMP9 signalling plays a role at the tumour–stroma border.

### Blockade of MAPK7/MMP signalling axis suppresses monocyte infiltration, TAM accumulation, tumourigenesis and lung metastasis

**Fig. 6:**
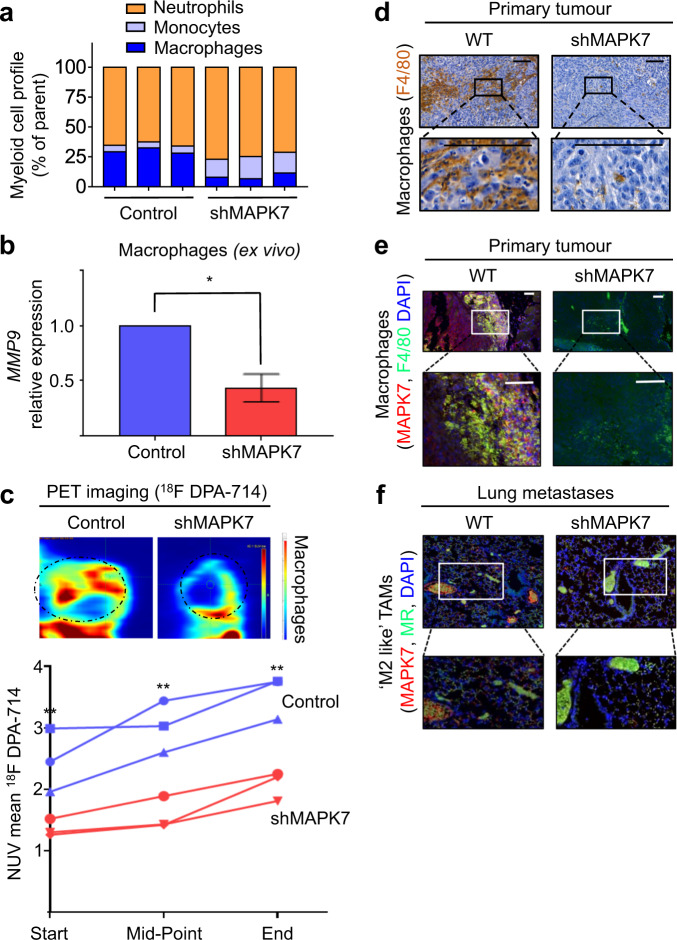
Blockade of MAPK7/MMP signalling axis suppresses TAM differentiation and lung metastasis. **a** FACS analysis of the immune profiles of control and shMAPK7 tumours. Immune profiles are normalised to the total CD45^+^ cells (% of parent myeloid cells) in each sample to enable direct comparison between groups. We show immune profiles from three representative control and shMAPK7 tumours. Data are presented as the percentage of parent: myeloid cells. **b** qPCR analysis of macrophages isolated from tumours. *MMP9* mRNA was normalised to *ACTB*. Macrophages from shMAPK7 tumours have significantly less *MMP9* expression suggesting tumour MAPK7 signalling regulates macrophage *MMP9* expression. **c** Positron emission tomography (PET) imaging using ^18^F DPA-714 tracer to detect intratumoural macrophage expression. Representative end point PET images are shown (heat map images). Tumours lacking *MAPK7* have fewer macrophages than size-matched control tumours, and unlike control tumours, they do not display an increase in macrophage influx over the course of tumour growth (graph) (*p* ≤ 0.001). **d** Chromogenic IHC analysis of tumour biopsies. shMAPK7 tumours display marked reduction in intratumoural macrophages (F4/80). **e** Fluorescent IHC analysis of tumour biopsies. shMAPK7 tumours have significantly fewer macrophages (F4/80). MAPK7 expression co-localises with macrophages in control tumours and is absent in shMAPK7 tumours. **f** Fluorescent IHC analysis of lung biopsies from tumour bearing animals. Few ‘M2-like’ TAMs are detected in the lungs of mice bearing shMAPK7 tumours compared to a strong infiltration of ‘M2-like’ TAMs in the lungs of animals bearing control tumours (MR). Lungs from control animals have greater MAPK7 expression when compared to the lungs of animals bearing shMAPK7 tumours. Together this shows a tumour MAPK7 signal controls both TAM infiltration and MAPK7 activity at the metastatic site (lung). Scale bars are 100 μM. Representative images are used to describe data collected from 12 mice per group. Data are mean ± SD of three biological replicates. *p ≤ 0.05, **p ≤ 0.001.

Previous work in skin and lung cancer shows that MAPK7 promotes pro-tumour inflammation plus ‘M2-like’ polarisation of tumour-associated macrophages (TAMs) [29, 36]. Since (i) recent evidence has shown that there is significant crosstalk between osteosarcoma and the immune system [14], (ii) *TP53*^−/−^ triggers WNT-dependent systemic inflammation that stimulates TAMs to perform breast cancer metastasis [37], and (iii) our data here in a *TP53*^−/−^-driven cancer that shows MAPK7 regulates *MMP9* and is involved in lung metastasis, we strongly suspected that MAPK7/MMP9-driven TAMs were mediators of osteosarcoma metastasis [14, 37]. We performed immunohistochemistry (IHC) plus cell sorting of immune cell composition in control and shMAPK7 tumours. Fluorescence-activated cell sorting (FACS) analysis showed a significant reduction in CD45+ tumour infiltrates, that is, there were fewer immune cells present in shMAPK7 tumours (data not shown). To directly compare immune cell constitution or ‘immune contexture’ between control and shMAPK7 tumours, we normalised immune cell numbers to the total number of CD45+ cells in each sample (Fig. 6a). The immune contexture in shMAPK7 tumours was composed of fewer macrophages, greater numbers of neutrophils plus a greater number of monocytes (Fig. 6a). We isolated macrophages from shMAPK7 tumours. These macrophages displayed an impaired ability to produce *MMP9* despite having intact *MAPK7* themselves (Fig. 6b). Non-invasive imaging using ^18^F DPA-714 to detect translocator protein (*TSPO*) expressing cells in vivo [38] that are predominantly macrophages showed a significant decrease (*p* ≤ 0.01) in macrophage infiltration in shMAPK7 tumours (Fig. 6c). We next used a pan macrophage F4/80 marker to show that control primary tumours contained higher levels of macrophage infiltration when compared to shMAPK7 tumours (Fig. 6d). Macrophage-rich regions in control primary tumours co-localised with MAPK7 expression (Fig. 6e). ‘M2-like’ and MAPK7-expressing TAMs were almost completely absent in the lungs of mice with shMAPK7 tumours (Fig. 6f). These observations were despite the fact that macrophages and lung tissue have intact *MAPK7*. Together, these experiments show that a MAPK7 signal derived from primary tumour cells regulates TAM polarisation, TAM expression of *MMP9*, TAM infiltration and TAM-mediated metastasis to the lungs.

## Discussion

Complex human diseases such as cancer accompany widespread reprogramming of gene expression. A comprehensive understanding of the disease state requires not only the identification of DE genes, but also understanding the cellular and physiological responses to dysregulated expression patterns. Here our analyses allowed us to view the transcriptomic alterations that underpin PBC metastasis at whole tumour and single-cell resolution. We have uncovered several transcripts involved in PBC malignant progression that were undetected in previous genomic studies. Some of these genes and regulatory network hubs are clinically actionable with available drugs. Out of the significant amount of data generated here, we interrogated *MMP9* owing to its extremely high expression plus recurrent observation in our models. Experimental data on *MMP9* was independently achieved across two separate laboratories supporting our inference that *MMP9* is involved in PBC spread to the lungs. Our experiments showed that MAPK7 is an upstream master regulator of *MMP9* and is responsible for driving metastasis. This observation is consistent with in vitro models and tail vein injection metastasis models [30, 32, 33]. Here we modelled lung metastasis with markedly more biological and clinical significance because we tracked metastatic spread of human cancer that produced orthotopic tumours.

Tumour cells harbouring shMAPK7 showed impaired tumour growth compared to controls. No difference in proliferation was observed between control and shMAPK7 143B cells in vitro (Supplementary Fig. 2d). We hypothesised the delayed growth of shMAPK7 tumours in vivo was due to shMAPK7 tumours lacking the ability to effectively crosstalk with the stromal and immune compartments, which can accelerate tumour growth. We have shown in other studies that MAPK7 is a fundamental requirement for a pro-tumour immune contexture [28, 29].

We showed a MAPK7 signal and/or MAPK7 sensitivity is required for PBC metastatic spread to the lungs. Metastatic spread to other parts of the body, including other skeletal sites, was not observed in the timeframe of this study. To control for the slower growth rate of shMAPK7 tumours versus controls, we tested metastatic spread to the lung at equivalent tumour sizes in each cohort. The longer time taken for shMAPK7 tumours to reach equivalent size to their control counterparts increased the overall tumour residency time, which we know from several studies positively correlates with increased metastatic risk. This illustrates that the lack of development of lung metastases from shMAPK7 tumours is even more significant.

Previous work on MAPK7 has shown it to be a driver for epithelial–mesenchymal transition (EMT) [39]. PBC arises from and is itself a mesenchymal tissue. EMT is all but redundant in this context. We focussed our evaluation of driver mechanisms underpinning our observations on the immunological effects of *MAPK7* loss, as seen in other cancer models, and the interaction with MMP9 signalling therein.

Fundamental to several cancers is a specific macrophage population arising from blood monocytes. TAMs are perpetually recruited to tumours. In early tumours, TAMs present an inflammatory and tumoricidal ‘M1-like’ phenotype. As tumours progress, TAMs are functionally reprogrammed by tumour-derived signals to exhibit a trophic, angiogenic and immune inhibitory ‘M2-like’ phenotype that contributes to advancing cancer [29]. Here *MAPK7* silencing strongly minimises TAM infiltration at the tumour site while increasing the monocyte content. This finding supports the conclusion that MAPK7 controls TAM maturation and phenotype, which is also observed in other cancer types [29]. *MAPK7* loss affects macrophage residency and the lung phenotype of tumour bearing animals despite *MAPK7* loss only occurring in tumour cells. MAPK7 expression co-localises with TAMs at both the primary and secondary site. The lungs of mice bearing tumours lacking *MAPK7* have fewer ‘M2-like’ macrophages. These observations suggest that a tumour-derived MAPK7 signal supports the lung microenvironment to be conducive to metastases by supporting macrophage influx and by directing their polarisation to a pro-metastatic ‘M2-like’ phenotype. *MAPK7* loss in primary tumours decreases MAPK7 expression in the lungs, suggesting that MAPK7 regulates a positive feedback loop for its own expression between the primary and metastatic site. *MAPK7* loss in xenograft tumours reduces *MMP9* expression in TAMs that have intact *MAPK7*, further supporting our assertion that MAPK7 signals dictate TAM behaviour and phenotype. Taken together, this work shows that a tumour-derived MAPK7 signal dictates macrophage behaviour at the primary site plus secondary lesion to provide molecular cues for immune contexture and metastatic spread in PBC.

Owing to the prevalence of TAMs in solid cancer plus their unique influence on disease progression, macrophage-targeted interventions have attracted prominent attention in cancer immunotherapy. Amenable targets to reduce TAM polarisation and infiltration are few because the signalling mechanisms underpinning malignant macrophage phenotypes are largely unknown. Here we have investigated the role of the MAP protein kinase MAPK7 as a determinant of macrophage polarity. Our data strongly implicate that TAMs drive metastasis to the lung using MAPK7/MMP9 in an autocrine and paracrine fashion [40, 41]. Targeting *MAPK7* affects the downstream expression of several other genes as well as *MMP9* (Supplementary Fig. 2d). Simultaneously targeting a broad range of genes will likely be required for clinically effective outcomes, that is, targeting *MMP9* plus other metastatic contributors, which could be made possible by targeting *MAPK7*.

There are currently no effective MAPK7 inhibitors. Older generation inhibitors had significant off-target effects that accounted for their observed phenotypes, but were originally attributed to MAPK7 [42]. The newest generation of MAPK7 kinase inhibitors have little if any effect on transcription of cancer-promoting genes [42]. Recent work shows MAPK7 kinase inhibitors can paradoxically activate MAPK7 [43]. This recent discovery plus our own previous work with genetic models of *MAPK7* loss show that loss of all MAPK7 functions, that is, its catalytic function plus non-catalytic transcriptional function, is required to successfully target MAPK7 for therapeutic gain [28, 29, 44]. None of the available inhibitors are able to inhibit all aspects of MAPK7 function, but future drug development should focus on achieving this objective.

Analytical tools that mine quantitative measurements of mRNA to identify key regulatory interactions and/or signalling can provide an effective avenue for identifying previously unknown molecular mechanisms with critical functions in health and disease. These computational strategies must be paired with rigorous experimentation to functionally validate and characterise the putative physiological outcome. Using this approach, we have established the role of a MAPK7/MMP9 signalling axis in recruiting TAMs to PBC tumours to induce lung metastasis. Removing the MAPK7/MMP9 signalling axis by RNA interference suppressed tumour burden, metastatic spread and increased overall survival in animals by inhibition of TAM infiltration. Our findings provide new insights into the mechanisms of PBC metastatic progression mediated by TAMs that may advance the development of immune-based strategies. Our results also demonstrate the value of unbiased sequencing strategies, such as whole tumour plus single0ell RNA0sequencing that do not rely on prior knowledge of annotated regulatory programs. The approach here finds that blockade of MAPK7/MMP9 signalling may overcome current hurdles for targeting pathways that ultimately lead to metastatic lung nodule formation in a childhood cancer.

## Materials and methods

### Patient samples

The University of East Anglia Faculty of Medicine and Health Sciences Research Ethics Committee approved the collection and study of human samples (Reference: 2015/16 100 HT). We obtained patient material from the Royal Orthopaedic Hospital, Royal Papworth Hospital and the UCL Biobank (*n* = 21). We confirmed high-grade osteoblastic osteosarcoma at biopsy and at resection. All individuals provided written informed consent to donate blood/tissue to this study. We used publicly available datasets from the European Nucleotide Archive (https://www.ebi.ac.uk/ena) and combined with our patient series before processing through our bioinformatics pipeline (*n* = 9).

### CTC capture and imaging

We isolated CTCs from 7.5 ml whole blood in EDTA using the ClearCell FX (Biolidics). Cells were deposited in 10 ml resuspension buffer, centrifuged at 500 × *g* for 10 min, the supernatant was removed, and then 100 μl was transferred to a Nunclon plate (Thermo Fisher Scientific). For imaging live cells, CTCs were cultured in DMEM (Dulbecco’s modified Eagle’s medium) high glucose (Thermo Fisher Scientific) containing 10% (v/v) foetal bovine serum (FBS) (Sigma-Aldrich) and 1% (v/v) penicillin–streptomycin. We cultured for 5 days and maintained at 37 °C in 5% CO_2_. For single-cell RNA-sequencing, we manually picked CTCs under a microscope using a P10 pipette set to 1 μl and placed individual cells into 10 μl of lysis buffer. We stained live CTCs with Hoechst 33342 (Thermo Fisher Scientific), a cell surface vimentin monoclonal antibody (Abnova) and a CD45 monoclonal antibody (BD Biosciences). Blue fluorescence was excited at 365 nm and emission collected between 420 and 470 nm. Red fluorescence was excited at 558 nm and emission was collected through a 615 nm LP filter. Green fluorescence was excited at 400 nm and emission collected through a 525 nm LP filter. We imaged CTCs using an Axiovert 200 M microscope (Zeiss) with an Axiocam MRm CCD camera (Zeiss) under the control of AxioVision.

### Library preparation and next-generation sequencing

We extracted total RNA using the miRNeasy mini kit (Qiagen) according to the manufacturer’s instructions. We measured concentration and integrity on the NanoDrop 8000 Spectrophotometer (Thermo Fisher Scientific). RNA was stored at −80 °C. We used the NEBNext ultra II RNA library prep kit (New England Biolabs) and SMART-seq v4 ultra low input RNA kit (Takara) to generate libraries. We performed 150 bp PE sequencing on a HiSeq 2500 (Illumina).

### Bioinformatics

We converted fastq files to fasta. We used Trim Galore to remove adapter sequences and reads <20 nt. Trimmed reads were aligned to the human genome (v38) using HISAT2 [45]. Transcripts were download from GENCODE (v28) and Ensembl (v92). Count matrices for transcripts were created using Kallisto [46]. We determined DE transcripts using the DESeq2 package in R (v1.2.10) [47]. We selected DE mRNA according to log_2_ fold change ≥2, *p* ≤ 0.05 and false discovery rate (FDR) <5%.

### Alternative splicing analysis

We examined alternative splicing events from aligned BAM files using rMATS [48]. rMATS quantified exon/intron by inclusion junction counts and skipped exon/intron by skipping junction counts. The difference in inclusion level for each candidate splicing event was calculated using reads that map to the body of exons as well as splice junctions from control and tumour samples. Differentially spliced events were required to have an absolute difference in inclusion level >10% plus an FDR < 10%. We used rMATS2Sashimiplot and Sashimi plot for quantitative visualisation [49].

### Gene expression analysis

To validate sequencing datasets, we performed gene expression analysis using a modified PanCancer Pathways Panel (NanoString Technologies) comprising 800 genes, including 12 housekeeping genes (Supplementary File 4). We used an nCounter Digital Analyser (NanoString Technologies) to count the digital barcodes representing the number of transcripts. Raw counts were automatically normalised by the total counts of all the tested samples and housekeeping genes in order to compensate for variations introduced by experimental procedures. We averaged counts between replicates using the nSolver analysis software and log_2_ transformation. We used the most stringent method (mean ± 2 SD) to accept detected transcripts.

### Gene set enrichment in CTCs

We ranked sequencing reads confirmed by nanostring into a numerical expression list. We built a network of functional interactions between the genes using STRING (v11). The line colour connecting genes indicates the known and predicted interactions. Blue lines represent data from curated databases. Pink lines represent data from experiments. Green lines represent gene neighbourhoods. Black lines represent co-expressed genes.

### Weighted gene co-expression network analysis

WGCNA was used to generate unsigned co-expression networks in controls, primary tumours and metastatic tumours [21]. Transcripts with normalised counts (TPM) > 5 were used for the co-expression analysis. WGCNA clusters genes into network modules using topological overlap measure (TOM). TOM is a robust measure of network interconnectedness and measures the connection strength between two adjacent transcripts and all other transcripts in a network. Hierarchical clustering was used to group transcripts based on dissimilarity of transcript connectivity, which is defined as 1-TOM. We used the cutreeDynamic function to produce co-expression clusters. The minimum size of modules was 20 transcripts and were randomly colour labelled. An adjacency matrix was built by applying a power function (*β*) on the Pearson correlation matrix. The *β* was optimised to be 18 for balancing the scale-free property of the co-expression network and the sparsity of connections between transcripts. Intramodular connectivity of transcripts was used to identify hubs in the modules.

### Cell culture

We obtained 143B (osteosarcoma) cells from ATCC. We authenticated cells by STR profiling. We cultured cells in DMEM (Thermo Fisher Scientific) containing 10% (v/v) FBS (Sigma-Aldrich) and 1% (v/v) penicillin–streptomycin. We refreshed culture media every other day and maintained at 37 °C in a atmosphere of 5% CO_2_. Cells were regularly monitored for *Mycoplasma* infection by PCR. Similar passage number was used in biological replicates in vitro and for implants in vivo.

### Immunoblotting

We extracted proteins in radioimmunoprecipitation assay buffer containing protease and phosphatase inhibitors. We resolved extracts (30 μg) by sodium dodecyl sulfate-polyacrylamide gel electrophoresis and electrophoretically transferred to an Immun-Blot® PVDF membrane (Bio-Rad). Membranes were saturated in 3% non-fat dry milk or 3% bovine serum albumin (BSA) and probed overnight at 4 °C with antibodies (1:1000 dilution unless otherwise indicated) to MAPK7 (Cell Signalling, #3372), MMP9 (Abcam, #Ab38898) and ACTB (Sigma, #A5316). We detected immunocomplexes by enhanced chemiluminescence with immunoglobulin G (IgG)-coupled to horseradish peroxidase as the secondary anti-rabbit and anti-mouse antibodies (Abcam).

### Quantitative polymerase chain reaction

Total RNA was isolated from cells using TRIzol and the miRNeasy mini kit (Qiagen). We carried out complementary DNA synthesis as previously described [28]. We performed quantitative polymerase chain reaction using the SYBR green I core kit (Eurogentec). Human *MMP9*, 5′-GTACTCGACCTGTACCAGCG-3′, 5′-AGAAGCCCCACTTCTTGTCG-3′; mouse *Mmp9*, 5′-GCCGACTTTTGTGGTCTTCC-3′, 5′-CTTCTCTCCCATCATCTGGGC-3′; human *PGK1*, 5′-GAAGATTACCTTGCCTGTTGAC-3, 5′-GCTCTCAGTACCACAGTCCA-3′. PCR products were detected in the ABI PRISM® 7700 sequence detection system (Thermo Fisher Scientific). We analysed results using the 2^−ΔΔ*G*^ method. Gene expression was normalised to *PGK1* or *ACTB*.

### Enzyme-linked immunosorbent assay

We performed MMP9 enzyme-linked immunosorbent assay (ELISA) using several kits (R&D Systems) according to the manufacturer’s instructions. Plates were pre-coated with MMP9 antibody. Briefly, fresh media were collected from equal numbers of cells and centrifuged to remove debris. We centrifuged supernatants in Amicon tubes (Millipore). We incubated plates with samples plus serial dilutions of provided ELISA standards. Plates were washed and incubated with a horse radish peroxidase (HRP)-conjugated secondary antibody, followed by a further wash plus incubation with a colorimetric HRP-sensitive substrate. We measured absorbance of the samples at 450 and 540 nM using a UQuant plate reader (BioTek). Absorbance at 540 nM was deducted from that at 450 nM to correct for background signal. We generated standard curves from the serial diluted standards and concentrations of MMP9 in the samples extrapolated from the standard curve.

### Mice

The University of Manchester Animal Welfare and Ethics Committee approved animal experiments. Experiments were performed under licence in accordance with UK Home Office guidelines and under the Animals (Scientific Procedures) Act 1986. Eight to twelve-week-old CD1-*Foxn1nu* female mice were implanted with 0.02 ml of a 6 × 10^7^/ml suspension containing either control or shMAPK7 143B cells into the left femur. Mice were housed in a pathogen-free facility. Mice were killed using Schedule 1 procedures. A small region of fresh lung was tied off and excised for clonogenic analysis. We inflated remaining lungs with formalin. We removed tumours, bisected and half fixed in 10% formalin, quarter digested for FACS analysis and a quarter frozen in liquid nitrogen for immunoblot or RNA analysis. For in vivo analysis using similar animal models, it has been shown that to detect >30% reduction in primary tumour growth, experiments require five animals per group. To detect >30% change in metastases with 0.8 power and at *p* ≤ 0.05 statistical significance, experiments require eight animals per group. For imaging to detect >30% changes with 0.8 power and at *p* ≤ 0.05 statistical significance, experiments require ten animals. For ex vivo analyses, animal numbers required to assess the functional role of MAPK7 in tumour inflammation and metastases was previously predicted by power analysis with a minimum of three tumours taken from three biological replicates for all analytical techniques [28]. Animal experiments were powered based on the experimental analysis that required the largest mouse number (imaging). To accommodate a potential implant failure rate ~10%, 15 mice per group were used (*n* = 15 control, *n* = 15 shMAPK7). These animals were divided into three independent experiments (*n* = 5 control, *n* = 5 shMAPK7). These numbers provided the three biological replicates needed to power ex vivo analysis. Exclusion criteria were those animals that did not develop tumours. No randomisation or blinding was used when allocating animals to experimental groups.

### Plasmids

We used pLenti CMV Puro LUC (w168-1) (Addgene) [50]. We used a SMARTvector (Dharmacon) plasmid for shMAPK7. We used third-generation pMD2_VSVg plus packaging plasmids pRSV-Rev (Addgene) and pMDLg/pRRE (Addgene) for luciferase lentiviral transduction. We used second-generation pMD2G (VSV-G envelope) and p8.91 (HIV gag/pol) for shRNA plasmids.

### Bacterial transformation

Transformation was carried out according to the manufacturer’s instructions using MAX Efficiency Stbl2™-competent cells (Invitrogen). Briefly, 100 μl of Stbl2 cells were thawed on wet ice and then aliquoted into cold polypropylene tubes. One microlitre of solubilised plasmid DNA was added to competent cells and incubated on ice for 30 min. Cells were heat shocked in a water bath at 42 °C for 25 s. Cells were placed on ice for 2 min and then 0.9 ml of ambient temperature SOC medium (2% tryptone, 0.5% yeast extract, 8.6 mM NaCl, 20 mM KCl and 20 mM glucose) was added. Ligation reactions were shaken (60 min, 225 r.p.m., 30 °C) and then diluted 1:10 with the SOC medium. One hundred microlitres was spread onto pre-warmed LB agar plates with pre-added ampicillin (100 μg/ml). Agar plates were incubated overnight at 30 °C and then the colonies were picked and used to produce starter cultures.

### Plasmid starter cultures

Plasmid starter cultures were taken from plasmid glycerol stocks stored at −80 °C. Using a sterile pipette tip, a small amount of glycerol stock was scraped into 50 ml centrifuge tubes containing 5 ml LB broth plus ampicillin (100 μg/ml). The CMV Puro LUC plasmid was picked from single colonies grown up from bacterial transformations. Starter cultures were grown at 30 °C for 8 h at 225 r.p.m. Five millilitre starter cultures were transferred to 500 ml LB broth in conical flasks containing 100 μg/ml ampicillin and incubated overnight at 30 °C at 225 r.p.m. to obtain large amounts of plasmid DNA.

### DNA purification

Concentrated plasmid DNA was prepared using the Endofree plasmid mega kit (Qiagen) according to the manufacturer’s protocol. Briefly, bacterial cells were lysed and then cleared via a filter. Endotoxins were removed from the cleared lysate that was then loaded onto a binding column. RNA, protein and other impurities were removed by washing. Plasmid DNA was eluted in a high salt buffer. Plasmid DNA was concentrated and desalted by isopropanol precipitation and collected by centrifugation then resuspended in TE buffer.

### High titre lentiviral vectors

To generate high titre lentivirus, we plated 1.5 × 10^6^ HEK 293T cells on 150 mm dishes (Corning) containing 16.5 ml antibiotic-free complete media and incubated overnight to adhere. For luciferase expression, cells were transfected the following day with the expression plasmids CMV Puro LUC, pMD2_VSVg, pRSV-Rev and pMDLg/pRRE in a 2:1:2:1 ratio. For MAPK7 knockdown, cells were transfected the following day with expression plasmids pMD2G and p8.91 in a 3:1 ratio. Plasmids were diluted in 150 mM NaCl (3 ml per plasmid) in a 50 ml Falcon (Corning). Three millilitres of polyethylenimine (PEI)/NaCl solution (1:12 ratio of 15 mM PEI:150 mM NaCl) was added dropwise to each plasmid dilution and incubated for 10 min at room temperature and then the plasmid/PEI solution was evenly distributed dropwise at 2 ml per plate. Twelve hours post transfection, media were aspirated and replaced. Forty eight hours post transfection, viral supernatant was aspirated and collected in 50 ml Falcon tubes, while fresh complete media were added to the plates. Falcons were centrifuged (5 min, 112 × *g*, 4 °C) to remove cell debris and filtered through a pre-wet 0.45 μm cellulose acetate filter (Corning) using a vacuum pump. The supernatant was then transferred into 50 ml Falcon tubes able to withstand high-speed centrifugation (Alpha Laboratories). Falcons were then centrifuged (2.5 h, 13,500 × *g*, 4 °C) to obtain viral pellets. The supernatant was aspirated and pellets resuspended in 100 μl formulation buffer (phosphate-buffered saline (PBS), 1 mg/ml human serum albumin, 5 μg/ml protamine sulphate), aliquoted and stored at −80 °C. A second harvest was conducted using the same protocol 72 h post transfection.

### Viral titre determination

We seeded 143B cells at 1 × 10^5^ cells per well in 12-well plates and left to adhere overnight. The following day, cells in one well were counted and then the remaining wells were infected with serial dilutions of lentiviral vector (10^−3^–10^−5^ per 1 ml medium). Media were changed after ~12 h, and then after 48-h incubation (37 °C, 5% CO_2_), cells were detached and transferred to microcentrifuge tubes. For luciferase titre determination, cell pellets were resuspended in 100 µl 4% paraformaldehyde and incubated at room temperature for 20 min. Fixed cells were resuspended in permeabilisation buffer (PBS, 0.5% BSA, 0.1% Triton X) for 10 min and then stained 1:200 for luciferase expression with anti-firefly luciferase antibody (Abcam) in FACS buffer (PBS, 0.5% BSA) for 30 min at room temperature. After primary staining, cells were stained 1:1000 with Alexa Fluor® 488-conjugated goat anti-mouse IgG secondary antibody in FACS buffer. TOPRO-3 was diluted 1:1000 in FACS buffer and 3 µl was added to each sample to determine cell viability. shRNA-infected cells were sorted live by green fluorescent protein expression. All samples were sorted on the FACS Canto II flow cytometer and analysed using the FACSDiva software (BD Biosciences).

### Lung clonogenic assay

Fresh lung pieces were digested using Liberase reagent 1 U/ml (Promega) supplemented with DNase 100 U/ml (Sigma) for 30 min at 37 °C with mild agitation. We passed cell digests through a cell strainer and the resultant single-cell suspension was centrifuged for 2 min at 1400 r.p.m. We plated cells at serial dilutions in six-well plates and grew in conditions favouring tumour cell growth, that is, 2 weeks in RPMI media containing 10% FBS and 1% glutamine. Colonies formed from tumour cells resident in the lung were fixed with 70% ethanol and stained with 1% methylene blue (Sigma). We blind counted positive colonies and were expressed as the number of colonies per milligram of lung tissue from which they originated.

### Bioluminescence and fluorescence imaging

For bioluminescence, mice received an intraperitoneal injection (150 mg/kg) of VivoGlo™ Luciferin (Promega) 5 min before imaging. For fluorescence, mice received an intravenous injection (2 nmol per mouse) of MMPSense™ 750 FAST (PerkinElmer) 18 h before imaging. Signals plus grey-scale photographic images were acquired using a Photon Imager™ (Biospace) and M3 Vision (Biospace). We maintained animals under general anaesthesia with 1–2% isoflurane, plus warming and monitoring, during image acquisition. We carried out signal quantification (photons/s/cm^2^/sr) using M3 Vision (Biospace).

### IHC (fluorescent and chromogenic)

We immunostained 5-μm-thick tissue sections with antibodies to MAPK7 (Cell Signalling, #3372, 1:200 dilution), F4/80 (Abcam, #Ab6640, 1:100 dilution), MMP9 (Abcam, #Ab38898, 1:200 dilution) and mannose receptor (MR) (1:1000 custom made). The reaction was revealed using either Vectastain ABC system (Vector Labs), followed by DAB (Vector Labs) and counterstained with haematoxylin (chromogenic IHC) or by fluorescence-conjugated Alexa Fluor secondary antibodies (Abcam, #A21054, #Ab150080, 1:1000) counterstained via DAPI (4′,6-diamidino-2-phenylindole) mounting medium (Abcam) (fluorescent IHC).

### Fluorescence-activated cell sorting

We generated cell suspensions from fresh tumour biopsies using Liberase reagent 1 U/ml (Promega) supplemented with DNase 100 U/ml (Sigma). Mononuclear single-cell suspensions were analysed by FACS. Briefly, cells were pelleted, washed twice and suspended in FACS solution (PBS containing 10% FBS). Cells were incubated for 30 min at 4 °C before being stained with the following antibody fluorophore conjugates: F4/80-P610 (Miltenyi, #130-107-709), CD11b-BUV661 (BD, #565080), CD45-A700 (BD, #565478), CD3-PeCy7 (BD, #560591), CD4-PCP Cy5.5 (BD, #561115), CD25-421 (BD, #564571), CD127-PE (BD, #562419), Ly6C-APC cy7 (BD, #128026) and Ly6G-AF-488 cy5.5 (BioLegend, #127625). Compensation bead analysis was used to define fluorescence channel parameters. We assessed cell viability by DAPI (Molecular Probes) to discriminate dead from live cells. We performed flow cytometry with a FACScan (BD) and analysed using the FlowJo software. Data were generated as % of parent: myeloid cells. FACS gating strategy is shown in Supplementary Fig. 3.

### Positron emission tomography (PET)

Mice underwent dynamic baseline scanning when tumour size had reached ~200 mm^3^. We anaesthetised mice with 1–2% isoflurane. We catheterised the tail vein and placed mice in animal beds, that is, Minerve small animal environment system (Bioscan). We transferred beds to a preclinical PET/computed tomography scanner (Siemens). At the start of the acquisition, mice were injected with ~10 MBq of ^18^F DPA-714. We collected list mode data for 1 h. We maintained anaesthesia during image acquisition via a nose cone with respiration and temperature monitored throughout. Mice recovered in a warmed chamber after imaging. We re-scanned mice at 14 and 28 days after the start of treatment.

### Image reconstruction and data analysis

Before image reconstruction, the list mode data were histogrammed with a span of three and maximum ring differences of 79 into 3D sinograms with 19 time frames (5 × 60 s, 5 × 120 s, 5 × 300 s, 3 × 600 s). We reconstructed images using the 3DOSEM and MAP algorithm (four OSEM3D iterations plus no MAP iterations with a requested resolution of 1.5 mm). We manually drew regions of interest (ROIs) over tumour, bone and contralateral bone as a reference using Inveon Research Workplace (Siemens). We performed further normalisation using the injected dose from the dose calibrator and mice weight to give a standardised uptake value (SUV). We calculated SUV mean as the average over all voxels within the ROI. We calculated normalised uptake value by dividing the SUV mean from the tumour and the tumour bearing bone from the contralateral bone. We performed normalisations in case the treatment caused systemic effects that would modify tracer uptake in healthy tissue.

### Statistical analysis

We evaluated variability between sequencing libraries using scatter plots, size-split box plots of the replicate-to-replicate differential expression, intersection and Jaccard similarity analysis [51]. Empirical differential expression was confirmed by parametric (*t*) and non-parametric (Mann–Whitney *U*, Wilcoxon’s signed-rank) tests. Differences in PET signal over time and sample tested were confirmed with two-way analysis of variance. For all statistical tests, we considered *p* ≤ 0.05 as statistically significant. All data presented in Figs. 1–3 passed log_2_ fold change ≥2, *p* ≤ 0.05 and FDR ≤ 5% parameters.

## Supplementary information

legends

Suppl. Fig. 1

Suppl. Fig. 2

Suppl. Fig. 3

Suppl. File 1

Suppl. File 2

Suppl. File 3

Suppl. File 4

## Data Availability

All data supporting the findings of this study are available within the article and Supplementary files or from the corresponding authors on request. Raw sequencing files are available at Gene Expression Omnibus (www.ncbi.nlm.nih.gov/geo) under the accessions GSE55282, GSE87624 and GSE140131.
