# Supplementary material for: Targeting the MAPK7/MMP9 axis for metastasis in primary bone cancer
Source: Oncogene. 2020 Jul 13;39(33):5553–69. doi: 10.1038/s41388-020-1379-0 (PMC7426263; doi:10.1038/s41388-020-1379-0)
Supplement: Supplementary file 1 — legends [file 41388_2020_1379_MOESM1_ESM.docx]

**FIGURE LEGENDS**

**Fig. 1.** Heat map based hierarchical cluster analysis of DE genes (x-axis) across tissue type (y-axis). Z score refers to high (red) and low (blue) gene expression using normalised values when compared to the mean of total sequencing reads. Pie charts below each heat map visually represent altered genes/pathways. **a.** Control bone versus primary tumour. **b.** Control bone versus metastatic lesion. **c.** Primary tumour versus metastatic lesion. There were few differences in gene expression between MAP treated and non-MAP treated patients. Patients are presented as one cohort, which will also include endogenous genetic heterogeneity. Each transcript presented has passed log_2_ fold change ≥2, *p* = <0.05 and FDR ≤5% parameters.

**Fig 2. a.** CTCs are positive for cell surface vimentin and negative for CD45. Scale bar is 50 uM. **b.** Pie chart visually represents the most enriched transcripts. **c.** Pie chart visually represents the least enriched transcripts. **d.** Gene-gene connections at high confidence (scores between 0.7 and 0.9). Line colour connecting genes indicates the known and predicted interactions. Blue lines represent data from curated databases. Pink lines represent data from experiments. Green lines represent gene neighbourhoods. Black lines represent co-expressed genes.

**Fig. 3. a.** Biplot principle component analysis (PCA) shows groups along the PC1 axis that correspond to primary (blue triangles) and metastatic (green circles) PBC plus controls (red crosses). **b.** WGCNA cluster dendrogram on all samples groups genes into distinct driver modules. Co-expression distance (TO, topology overlap) between genes (y-axis) and to genes (x-axis). Gene modules are colour coded. We selected the Green module where *E2F1* is a hub gene for further analysis because of its relationship to *TP53* and that *MMP9* was a component of the module. **c.** Heat map based hierarchical cluster analysis of the Green module show clear and distinct expression patterns between tissue types. Z score refers to high (red) and low (blue) gene expression using normalised values when compared to the mean of total sequencing reads. **d.** Gene-gene connections for the Green module. **e.** GO analysis using REVIGO^37^ scatterplot visualisation shows the cluster representatives in a two dimensional space derived by applying multidimensional scaling to a matrix of the GO terms’ semantic similarities. Bubble colour indicates *p* value. Bubble size indicates the frequency of the GO term in the underlying gene ontology annotation GO term database. **f.** Molecular functions significantly affected.

**Fig. 4. a.** Immunoblot analysis of MAPK7 expression in 143B cells demonstrating knockdown efficiency of shMAPK7 lentiviral preparations E1, E2 and E3. E2 induced the greatest decrease in MAPK7. E2 mediated MAPK7 cells were used for the rest of the study and are referred to as shMAPK7 cells hereafter. **b.** Immunoblot analysis of MMP9 demonstrating loss of MMP9 expression following MAPK7 knockdown in 143B cells. **c.** qPCR analysis showing MAPK7 knockdown induces a significant decrease in *MMP9* mRNA. *MMP9* mRNA levels were normalised to *PGK1* mRNA. **d.** ELISA analysis of culture media demonstrates that loss of MAPK7 significantly reduces MMP9 secretion by 143B cells. **e.** Bioluminescence imaging (BLI) to measure tumour burden in mice implanted intrafemorally with control and shMAPK7 143B cells. Tumours derived from shMAPK7 cells have delayed growth compared to control and display no detectable metastatic spread to the lung (absence of BLI signal in lungs of animals harbouring shMAPK7 143B tumours). **f.** Tumour H&E stain from control and shMAPK7 tumours and lungs. **g.** Lung clonogenic assay to detect micro metastatic spread to the lung. Lungs from mice harbouring shMAPK7 tumours had virtually no lung clonogenicity (*p* = <0.001). Representative images are used to describe data collected from 12 mice per group. Data are mean ±SD of three biological replicates.

**Fig. 5. a.** Fluorescence imaging to detect active MMP9 in tumours *in vivo*. Tumours lacking MAPK7 had no detectable MMP9 activity. Images are from size matched control and shMAPK7 tumours. **b.** Quantified FLI signal in tumours over time. FLI signal indicative of MMP9 activity increases over time in control but not shMAPK7 tumours. **c.** IHC analysis of tumour biopsies. shMAPK7 tumours display marked reduction in MAPK7 expression but still display MAPK7 positive cells in the stroma (arrow). MMP9 expression was observed at the leading edge of control tumours (arrow) but was undetectable in shMAPK7 tumour biopsies. Scale bar is 100 uM. **d.** qPCR analysis of *ex vivo* tumour lysates. shMAPK7 tumours have significantly less *MMP9* mRNA expression. *MMP9* mRNA levels were normalised to *PGK1* (*p* = <0.001). Representative images are used to describe data collected from 12 mice per group. Data are mean ±SD of three biological replicates.

**Fig. 6.** **a**. FACS analysis of the immune profiles of control and shMAPK7 tumours. Immune profiles are normalised to the total CD45+ cells (% of parent myeloid cells) in each sample to enable direct comparison between groups. We show immune profiles from three representative control and shMAPK7 tumours. Data is presented as percentage of parent: myeloid cells. **b.** qPCR analysis of macrophages isolated from tumours. *MMP9* mRNA was normalised to *ACTB*. Macrophages from shMAPK7 tumours have significantly less *MMP9* expression suggesting tumour MAPK7 signalling regulates macrophage *MMP9* expression. **c.** Positron emission tomography (PET) imaging using ^18^F DPA-714 tracer to detect intratumoural macrophage expression. Representative end point PET images are shown (heat map images). Tumours lacking *MAPK7* have fewer macrophages than size matched control tumours and unlike control tumours do not display an increase in macrophage influx over the course of tumour growth (graph) (*p* = <0.001). **d.** Chromogenic IHC analysis of tumour biopsies. shMAPK7 tumours display marked reduction in intratumoural macrophages (F4/80). **e.** Fluorescent IHC analysis of tumour biopsies. shMAPK7 tumours have significantly fewer macrophages (F4/80). MAPK7 expression co-localises with macrophages in control tumours and is absent in shMAPK7 tumours. **f.** Fluorescent IHC analysis of lung biopsies from tumour bearing animals. Few ‘M2 like’ TAMs are detected in the lungs of mice bearing shMAPK7 tumours compared to a strong infiltration of ‘M2 like’ TAMs in the lungs of animals bearing control tumours (MR). Lungs from control animals have greater MAPK7 expression when compared to the lungs of animals bearing shMAPK7 tumours. Together this shows a tumour MAPK7 signal controls both TAM infiltration and MAPK7 activity at the metastatic site (lung). Scale bars are 100 uM. Representative images are used to describe data collected from 12 mice per group. Data are mean ±SD of three biological replicates.

**Suppl. Fig. 1.** Sashimi plots show alternatively spliced exons and flanking exons in representative samples. Per base expression is plotted (y-axis) with genomic coordinates (x-axis). Arcs represent splice junctions connecting exons and display the number of reads split across the junction (junction depth). mRNA isoforms are shown underneath each sashimi plot (exons in black squares with grey lines, introns as black lines). **a.** *OGFOD2* is an example to show skipped exon events but the transcript also displays three other events. **b.** *ATGD4* shows retained intron events but the transcript also displays three other events. **c.** *TPM1* shows 5’ alternative splice site events but the transcript also displays three other events. **d.** *TMEM218* shows 3’ alternative splice site events but the transcript also displays three other events. **e.** *CPNE1* shows mutually exclusive events but the transcript also displays three other events. **f.** *WBP1* is an example sashimi plot to show massive disruption by at least four of five alternative splicing events in one transcript that also occurs in *OGFOD2*, *ATGD4*, *TPM1*, *TMEM218* and *CPNE1*.

**Suppl. Fig. 2. a.** MTT assay measuring cell proliferation *in vitro*. **b.** Exogenous expression of luciferase had no impact on cell proliferation rates. **c.** Luciferase activity assay *in vitro*. Luciferase activity in control versus shMAPK7 luciferase cells is equivalent. Bioluminescence signals (Fig. 4 and Fig. 5) are directly indicative of tumour growth rates *in vivo*. **d.** mRNA expression of oncogenic/metastatic genes in control cells or cells expressing constructs E1, E2 or E3 from Fig. 4. mRNA levels were normalised to control genes (*GAPDH* and *HPRT1*). *JNK1* is used as a negative control, i.e. is known to be unaffected by MAPK7 knockdown. Data are mean ±SD from three biological replicates (**a-c**). Data are from one biological replicate performed in triplicate (**d**).

**Suppl. Fig. 3.** FACS gating strategy.

**Suppl. File 1.** Patient samples.

**Suppl. File 2.** CTC data.

**Suppl. File 3.** WGCNA.

**Suppl. File 4.** Nanostring data.
