# Supplementary material for: Targeting the MAPK7/MMP9 axis for metastasis in primary bone cancer
Source: Oncogene. 2020 Jul 13;39(33):5553–69. doi: 10.1038/s41388-020-1379-0 (PMC7426263; doi:10.1038/s41388-020-1379-0)
Supplement: Supplementary file 2 — Suppl. Fig. 1 [file 41388_2020_1379_MOESM2_ESM.pptx]

## Slide 1
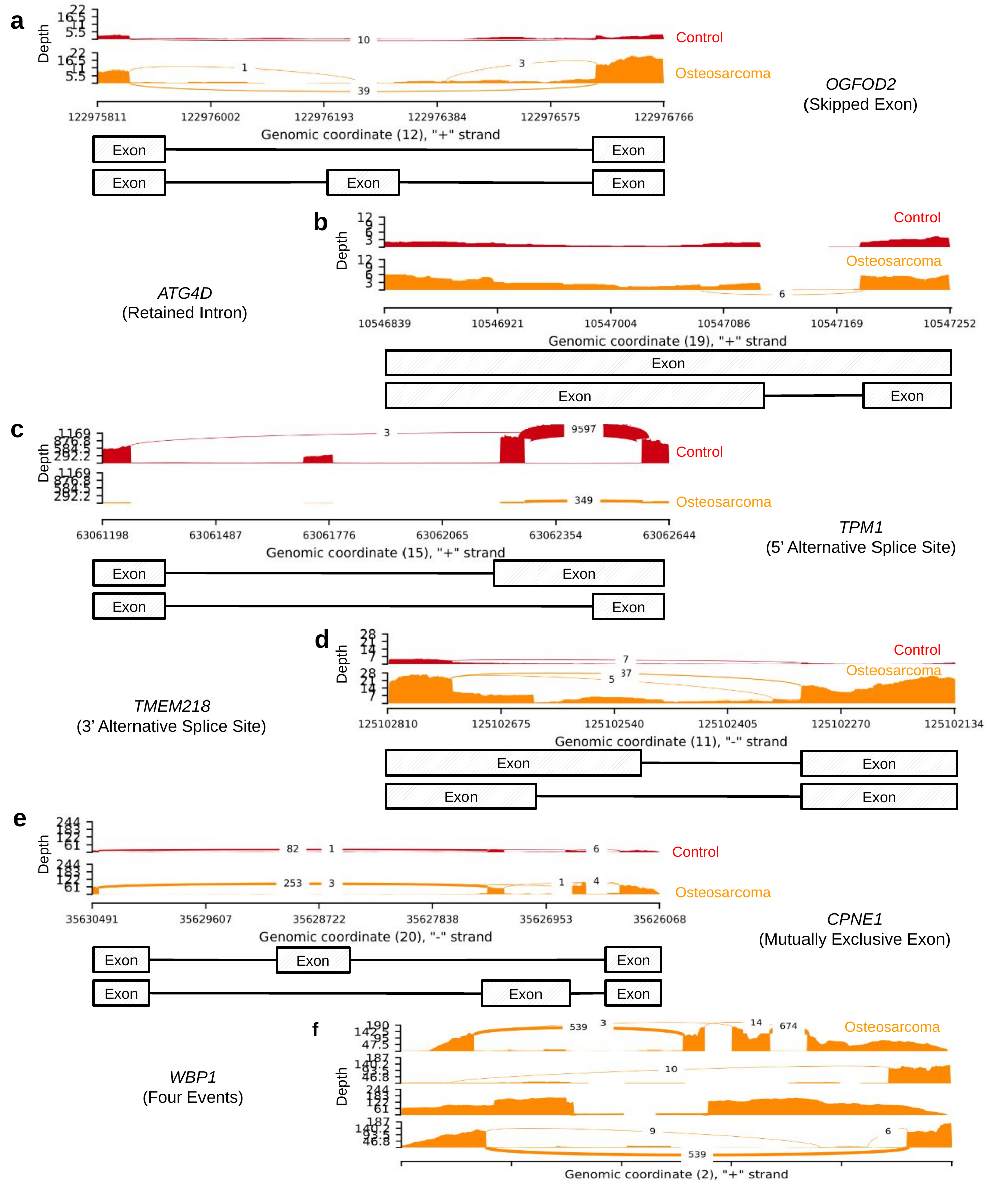

a
Control
Depth
Osteosarcoma
OGFOD2
(Skipped Exon)
Exon
Exon
Exon
Exon
Exon
b
Control
Depth
Osteosarcoma
ATG4D
(Retained Intron)
Exon
Exon
Exon
c
Control
Depth
Osteosarcoma
TPM1
(5’ Alternative Splice Site)
Exon
Exon
Exon
Exon
d
Control
Depth
Osteosarcoma
TMEM218
(3’ Alternative Splice Site)
Exon
Exon
Exon
Exon
e
Control
Depth
Osteosarcoma
CPNE1
(Mutually Exclusive Exon)
Exon
Exon
Exon
Exon
Exon
Exon
f
Osteosarcoma
WBP1
(Four Events)
Depth
