# Supplementary material for: Targeting the MAPK7/MMP9 axis for metastasis in primary bone cancer
Source: Oncogene. 2020 Jul 13;39(33):5553–69. doi: 10.1038/s41388-020-1379-0 (PMC7426263; doi:10.1038/s41388-020-1379-0)
Supplement: Supplementary file 3 — Suppl. Fig. 2 [file 41388_2020_1379_MOESM3_ESM.pptx]

## Slide 1
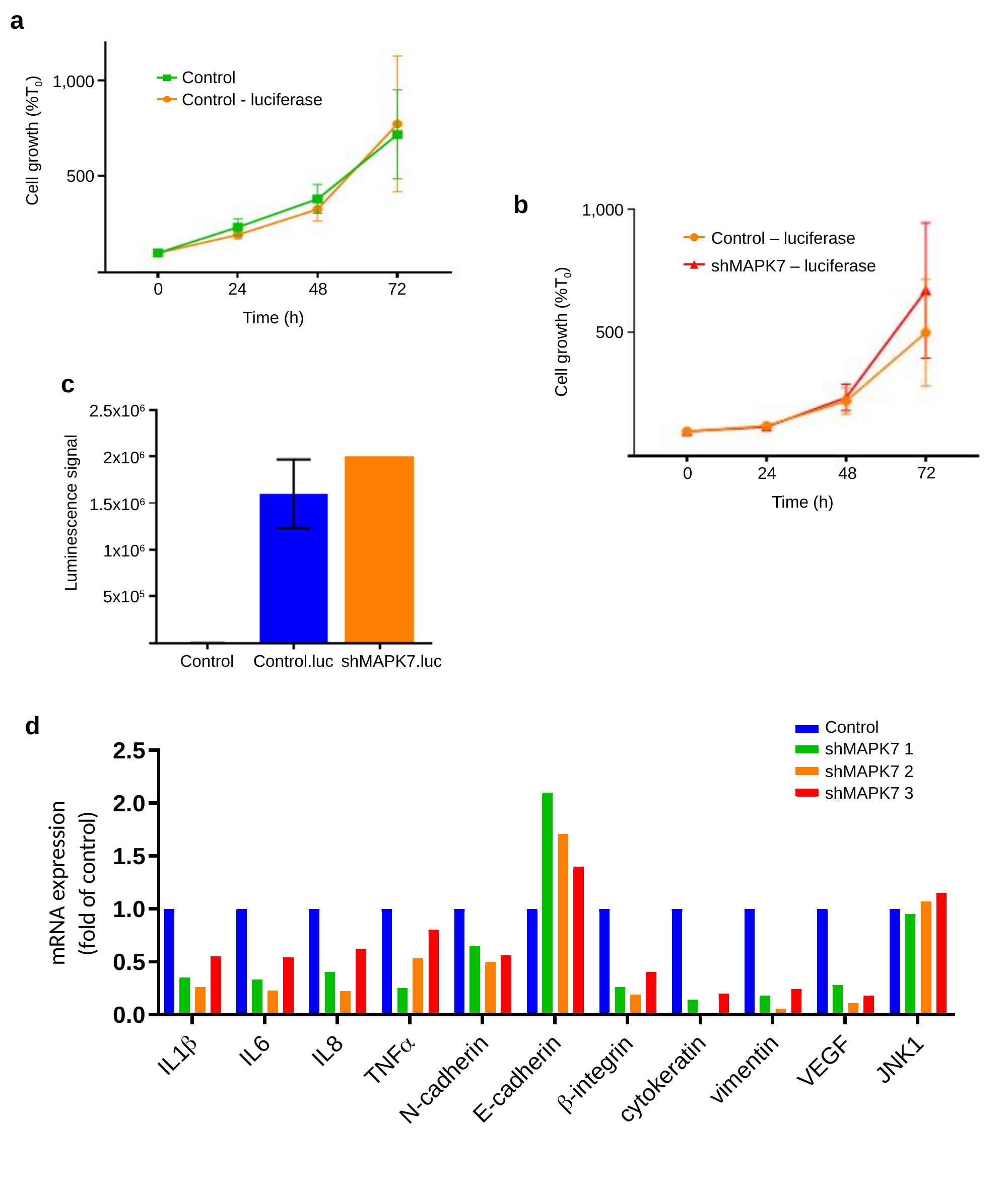

a
Control
1,000
Control - luciferase
Cell growth (%T0)
500
b
1,000
Control – luciferase
shMAPK7 – luciferase
72
0
24
48
Time (h)
500
Cell growth (%T0)
c
2.5x10^6^
2x10^6^
72
0
24
48
Time (h)
1.5x10^6^
Luminescence signal
1x10^6^
5x10^5^
Control
Control.luc
shMAPK7.luc
d
Control
shMAPK7 1
shMAPK7 2
shMAPK7 3
