# Supplementary figures and images for: Targeting the MAPK7/MMP9 axis for metastasis in primary bone cancer
Source: Oncogene. 2020 Jul 13;39(33):5553–69. doi: 10.1038/s41388-020-1379-0 (PMC7426263; doi:10.1038/s41388-020-1379-0)

## Slide 1
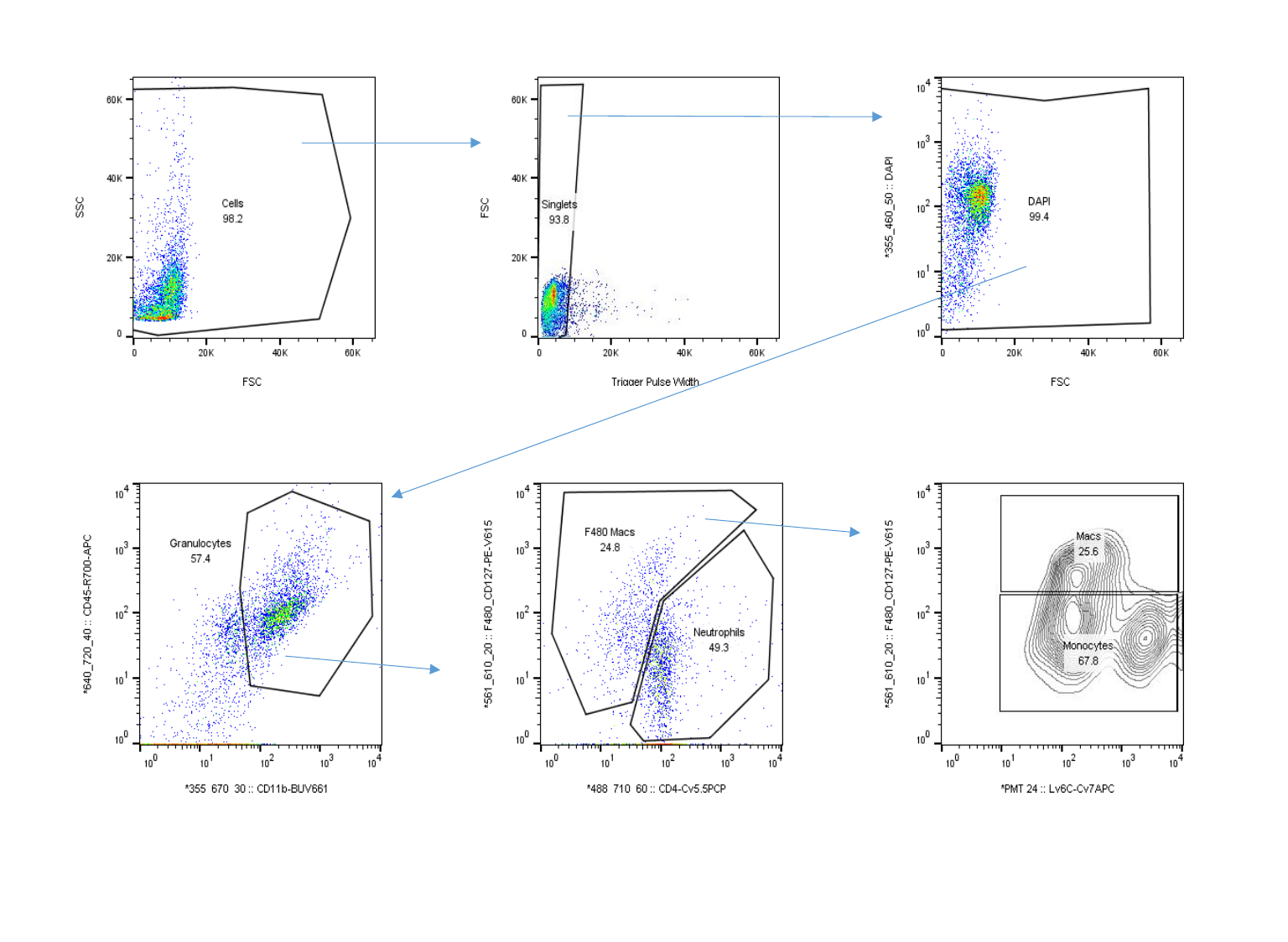

Supplement: Supplementary file 4 — Suppl. Fig. 3 [file 41388_2020_1379_MOESM4_ESM.pptx]
